# Quorum Sensing: A Prospective Therapeutic Target for Bacterial Diseases

**DOI:** 10.1155/2019/2015978

**Published:** 2019-04-04

**Authors:** Qian Jiang, Jiashun Chen, Chengbo Yang, Yulong Yin, Kang Yao

**Affiliations:** ^1^Laboratory of Animal Nutritional Physiology and Metabolic Process, Institute of Subtropical Agriculture, Chinese Academy of Sciences, China; ^2^University of Chinese Academy of Sciences, Beijing 100043, China; ^3^Department of Animal Science, University of Manitoba, Winnipeg, MB, Canada R3T 2N2

## Abstract

Bacterial quorum sensing (QS) is a cell-to-cell communication in which specific signals are activated to coordinate pathogenic behaviors and help bacteria acclimatize to the disadvantages. The QS signals in the bacteria mainly consist of acyl-homoserine lactone, autoinducing peptide, and autoinducer-2. QS signaling activation and biofilm formation lead to the antimicrobial resistance of the pathogens, thus increasing the therapy difficulty of bacterial diseases. Anti-QS agents can abolish the QS signaling and prevent the biofilm formation, therefore reducing bacterial virulence without causing drug-resistant to the pathogens, suggesting that anti-QS agents are potential alternatives for antibiotics. This review focuses on the anti-QS agents and their mediated signals in the pathogens and conveys the potential of QS targeted therapy for bacterial diseases.

## 1. Introduction

Antibiotics have been commonly used to prevent bacterial infection and diseases for many decades since their discovery at the beginning of the 20^th^ century. However, emerging evidence [1–6] indicates that traditional antibiotic treatments tend to be ineffective for the patients, due to the emergence of drug-resistant pathogens resulting from antibiotics overuse [7, 8]. The fact that bacterial infection annually deprives about 16 million human lives prompts us to develop novel approaches fighting against the drug-resistant pathogens and related diseases [9].

Bacterial quorum sensing (QS) signaling can be activated by the self-produced extracellular chemical signals in the milieu. The QS signals mainly consist of acyl-homoserine lactones (AHLs), autoinducing peptides (AIPs) and autoinducer-2 (AI-2), all of which play key roles in the regulation of bacterial pathogenesis. For instance, studies [10–12] reported that QS signals participate in the synthesis of virulence factors such as lectin, exotoxin A, pyocyanin, and elastase in the* Pseudomonas aeruginosa* during bacterial growth and infection. The synthesis and secretion of hemolysins, protein A, enterotoxins, lipases, and fibronectin protein are regulated by the QS signals in the* Staphylococcus aureus* [13, 14]. These virulence factors regulated by QS help bacteria evade the host immune and obtain nutrition from the hosts.

The anti-QS agents, which are considered as alternatives to antibiotics due to its capacity in reducing bacterial virulence and promoting clearance of pathogens in different animal model, have been verified to prevent the bacterial infection. The clinical application of anti-QS agents is still not mature. This review builds on the increasing discoveries and applications of the anti-QS agents from the studies in the past two decades. Our goal is to illustrate the potential of exploiting the QS signals-based drugs and methods for preventing the bacterial infection without resulting in any drug-resistance of pathogens.

## 2. Quorum Sensing Signals

The bacterial QS signals mainly consist of acyl-homoserine lactones (AHLs), autoinducing peptides (AIPs), and autoinducer-2 (AI-2) and participate in the various physiological processes of bacteria including biofilm formation, plasmid conjugation, motility, and antibiotic resistance by which bacteria can adapt to and survive from disadvantages [15]. The Gram-negative and Gram-positive bacteria have different QS signals for cell-to-cell communications. The AHL signaling molecules are mainly produced by Gram-negative bacteria [16], and AIP signaling molecules are produced by the Gram-positive bacteria [17]. Both Gram-negative and Gram-positive bacteria produce and sense the AI-2 signals [18]. These three families of QS signals are gaining more and more attention due to their regulatory roles in bacterial growth and infection.

Lux-I type AHL synthase circuit has been considered as the QS signals producer in the Gram-negative bacteria [19]. Once the AHLs accumulate in the extracellular environment and exceed the threshold level, these signal molecules will diffuse across the cell membrane [20] and then bind to specific QS transcriptional regulators, thereby promoting target gene expression [21]. The signal molecules AIPs are synthesized in Gram-positive bacteria and secreted by membrane transporters [17]. When an environmental concentration of AIPs exceeds the threshold, these AIPs bind to a bicomponent histidine kinase sensor, whose phosphorylation, in turn, alters target gene expression and triggers related physiological process [22]. For instance, QS signals in* Staphylococcus aureus* are strictly regulated by the accessory gene regulator (ARG) which associated with AIPs secretion [23, 24]. ARG genes are involved in the production of many toxins and degradable exoenzymes [25], which are mainly controlled by P2 and P3 promoters [26, 27]. The AGR genes also participate in the encoding of AIPs and the signaling transduction of histidine kinase [28]. Bacteria can sense and translate the signals from other strains in the environment known as AI-2 interspecific signals. AI-2 signaling in most bacterial strains is catalyzed by LuxS synthase [29, 30]. LuxS is involved not only in the regulation of the AI-2 signals but also in the activated methyl cycle and has been revealed to control the expressions of 400 more genes associated with the bacterial processes of surface adhesion, movement, and toxin production [31].

## 3. Biofilm Formation and Virulence Factors

Bacteria widely exist in the natural environment, on the surface of hospital devices, and in the pathological tissues [32]. Biofilm formation is one of the necessary requirements for bacterial adhesion and growth [33]. The biofilm formation is accompanied by the production of extracellular polymer and adhesion matrix [34, 35] and leads to fundamental changes in the bacterial growth and gene expression [36]. The formation of biofilm significantly reduces the sensitivity of bacteria to antibacterial agents [37, 38] and radiations [39] and seriously affects public health. Some formidable infections are associated with the formation of bacterial biofilms on the pathological tissues, and most infections induced by hospital-acquired bloodstream and urinary tract are caused by biofilms-coated pathogens on hospital medical devices. A large number of studies [33, 40, 41] have shown that bacterial quorum sensing (QS) signaling plays important roles in biofilm formation. Specific QS signaling blockage is considered an effective means to prevent the biofilms formation of most pathogens, thereby increasing the sensitivity of pathogens to antibacterial agents and improving the bactericidal effect of antibiotics [42, 43].

The production of virulence factors, which could help bacteria evade the host's immune response and cause pathological damage, is crucial for the pathogenesis of infections [44–46]. The virulence factors produced by different strains are different. For example, Gram-negative* Pseudomonas aeruginosa* produces virulence factors, such as pyocyanin, elastase, lectin, and exotoxin A [47, 48], and Gram-positive* Staphylococcus aureus *produces virulence factors such as fibronectin binding protein, hemolysin, protein A, lipase, and enterotoxin [49, 50]. Studies have shown that the production of these virulence factors is regulated by the bacterial QS signaling systems [51, 52]. Disruption of QS to control the production of virulence factors seems to be an attractive broad-spectrum therapeutic strategy.

## 4. Strategies for QS Disruption

The fact pathogens colonized in the host must active the QS signaling to form biofilm and produce virulence factors suggests that breaking this bacterial "conversation" by anti-QS agents makes pathogens more susceptible to host immune responses and antibiotics. In this section, we discuss the QS disruption strategies including receptor inactivation, signals synthesis inhibition, signals degradation, signaling blockage by antibody, and combining use with antibiotics and convey the potential of QS as the therapeutic target for bacterial diseases.

### 4.1. QS Receptor Inactivation

Inactivation of receptors in QS signaling is an effective strategy for reducing bacterial virulence and infection (Table 1). Studies [53] have demonstrated that flavonoids can bind to QS receptors and significantly reduce the virulence gene expression in* Pseudomonas aeruginosa*. N-decanoyl-L-homoserine benzyl ester, a structural analog of AHL signals, has been revealed to reduce the production of virulence factors, such as elastase and rhamnolipid, by blocking the homologous receptors in* Pseudomonas aeruginosa *[54, 55]. Receptor antagonists have been revealed to enhance the antibacterial activity of various antibiotics and minimize the therapeutic dose of antibiotics for* Pseudomonas aeruginosa* infection [56]. The meta-bromo-thiolactone was reported to prevent* Pseudomonas aeruginosa *infection by decreasing the pyocyanin production and inhibiting the biofilm formation [57]. Geske et al. have developed AHLs analogs that can bind with the LuxR, TraR, and LasR receptors in* Vibrio fischeri, Agrobacterium tumefaciens, and Pseudomonas aeruginosa*, respectively [58]. However, the application of receptor inhibitors for treating bacterial diseases is lagging behind due to the properties of instability and degradability within alkaline conditions. Further studies are warranted to improve the stability of these effective anti-QS agents.

### 4.2. QS Signals Synthesis Inhibition

The acyl-homoserine lactone molecules (AHLs) not only participate in bacterial communication but also play roles in conversations with eukaryotic cells. AHLs can regulate the signaling pathways in epithelial cells and affect the behavior of innate immune cells [59, 60]. Inhibiting the synthesis of AHLs is a direct strategy to reduce AHL-mediated virulence factors and prevent pathological damage (Table 2). For example, studies have revealed that the sinefungin, butyryl-SAM, and S-adenosylhomocysteine can attenuate the secretion of QS-mediated virulence factors and prevent the bacterial infection by inhibiting the AHLs synthesis in* Pseudomonas aeruginosa *[61–63]. Singh et al. reported that immucillin A and its derivatives can reduce the AHLs synthesis by inhibiting the 5-MTAN/S-adenosylhomocysteine nucleosidase [64]. The triclosan has been verified to reduce AHL synthesis by inhibiting the production of enoyl-ACP reductase precursors [65, 66]. However, these agents for AHLs synthesis inhibition also block the metabolism of amino acid and fatty acid that play key roles in bacterial basic nutrition [67]. The fact that triclosan increased the antibiotic-resistance of* Pseudomonas aeruginosa *implies selective pressure on bacteria were triggered by the blocking effects of triclosan on the metabolism of amino acid and fatty acid in the bacteria [68]. The triclosan is considered as bioindicator pollution due to its potential in causing the drug-resistance of the pathogens and increasing human health risks [69]. Thus, the drugs specifically targeting AHLs synthesis inhibition without blocking nutritional metabolisms of bacteria should be developed and identified by sufficient* in vitro *experiments before their clinical application.

### 4.3. QS Signals Degradation

Degradation of QS signals by enzymes can effectively disrupt the “communication” among the bacteria without causing any selective pressure to the bacteria. The enzymes consist of lactonase, acylase, oxidoreductases, and 3-Hydroxy-2-methyl-4(1H)-quinolone 2, 4-dioxygenase, all of which are derived from different bacterial strains and have been applied for QS signals degradation (Tables 3 and 4).

The AHL lactonase, a member of Metallo-*β*-lactamase superfamily, was able to prevent bacterial infection by degrading AHLs with different length of side chain [70, 71]. The AHL lactonases were reported to increase bacterial sensitivity to antibiotics without affecting the growth of* Pseudomonas aeruginosa *[72, 73] and* Acinetobacter baumannii *[74]. The AHL lactonase also has been applied to block the biofilm formation of* Pseudomonas aeruginosa *[75–77]. The AHL lactonase AiiK produced by the engineered* Escherichia coli* was revealed to inhibit extracellular proteolytic activity and pyocyanin production of* Pseudomonas aeruginosa* PAO1 [78]. In addition, synergistic action of AHL lactonase and antibiotics was observed in the mice model infected with* Pseudomonas aeruginosa*; that is, the drugs containing AHL lactonase can effectively inhibit the spread of skin pathogens while minimizing the effective dose of antibiotics. The AHL lactonase has also been applied in the fishery industry, for instance, Liu et al. reported the lactonase AIO6 supplemented to tilapia was able to prevent the* Aeromonas hydrophila* infection [79]. Studies reported the lactonase AiiA can decrease the virulence and inhibit biofilm formation of* Vibrio parahaemolyticus* in shrimps [80, 81].

The acylase, which was initially found in* Variovorax paradoxus* and* Ralstonia, *can block the QS signaling by hydrolyzing the amide bond of AHLs [82–84]. The acylase was revealed to decrease the growth of* Pseudomonas aeruginosa* ATCC 10145 and PAO1 by 60% [85, 86] and has been widely applied in human health care; for example, the acylase-coated device showed a well antibacterial property due to the QS signaling disruption by the acylase [87]. The acylase is also chemically immobilized on some nanomaterials to act as an antifouling agent [88]. Undoubtedly, these applications of acylase will greatly reduce the health care cost caused by the spread and colonization of pathogenic bacteria on medical devices.

Oxidoreductases are enzymes that can affect the AHLs specificity of homologous intracellular receptors by modifying acyl side chains, thus interfering with the expression of QS related virulence genes [89]. Previous studies have demonstrated the secretion of oxidoreductases by bacteria as a protective mechanism instead of a pathogenic signaling [90]. The BpiB09 oxidoreductase was reported to inhibit the activation of N-3-oxo-dodecanoyl homoserine lactone (3-oxo-C12-HSL) in the* Pseudomonas aeruginosa *PAO1 and decrease bacterial motility, biofilms formation, and pyocyanin secretion [91]. Immobilization of oxidoreductases on the glass surface can inhibit the bacterial biofilm formation and decrease the growth rate of* Klebsiella oxytoca* and* Klebsiella pneumoniae* [92, 93].

The dioxygenase has been revealed to block the quinolone signals in the QS system of* Pseudomonas aeruginosa* [94]. Dioxygenase can degrade 2-heptyl-3-hydroxy-4 (1H)-quinolone mediated signals and decreases signaling molecules accumulation in the bacterial milieu, therefore reducing the secretion of pyocyanin, rhamnolipid, and lectin A toxin, which protects the host from infective damage [95, 96].

Together, the anti-QS signaling enzymes are promising alternatives to antibiotics that can be used not only to control bacterial infection but also to minimize the risk of causing antibiotic-resistant strains. However, the stability of enzymes* in vivo* is the most difficult problem for their biomedical applications. It is of great significance to study and develop the stability of the anti-QS signaling enzymes* in vivo*. QS degradation by nonpathogenic bacteria is an effective strategy for QS disruption.* Pectobacterium carotovorum *subsp.* carotovorum *is a preferred and commonly used bacterial strain for QS degradation [97]. This biological strategy for QS signal degradation has been applied to prevent plant diseases [98] but has not been applied for human diseases treatment. By exploring novel QS-degradation strains, it might be possible to cure the chronic diseases caused by the antibiotic-resistant pathogens.

### 4.4. Target Antibodies for QS Blockage

The activation of AHL and AI-2 signaling can induce programmed cell death by affecting the host's immune system [59, 99]. Kaufmann et al. found in their study [100] that the antibody RS2-1G9 can bind to 3-oxo-C12-HSL in the extracellular environment of* Pseudomonas aeruginosa*, thereby attenuating the inflammatory response of the host. XYD-11G2 antibody has been shown to catalyze the hydrolysis of 3-oxo-C12-HSL signaling, thus inhibiting the pyocyanin production by Gram-negative bacteria [101, 102]. The monoclonal antibody AP4-24H11 was found to block the QS signal of Gram-positive* Staphylococcus aureus* by interfering with AIP IV [103]. Another* in vivo* study showed that the antibody AP4-24H11 could significantly attenuate the tissue necrosis in the infected model [104]. Although these monoclonal antibodies have been identified to block the QS signaling of pathogenic bacteria (Table 5), their applications for treating bacterial diseases are still in the initial stage.

### 4.5. Combinations of Anti-QS Agents and Antibiotics

Combining use of antibiotic with an anti-QS agent is the most effective clinical strategy for the treatment of bacterial diseases at present [105, 106]. Many studies have confirmed the synergistic effect of antibiotics and anti-QS agents (Table 6). Ajoene, furanone c-30, and horseradish extract have been revealed to reduce the expression of virulence factors in* Pseudomonas aeruginosa* and make* Pseudomonas aeruginosa* easier to be cleared by tobramycin [107–111]. Another study has confirmed the synergistic effects of curcumin, gentamicin, and azithromycin on* Pseudomonas aeruginosa*; that is, the expressions of virulence genes were significantly downregulated by the combining use of curcumin together with gentamicin or azithromycin, and the therapeutic doses of gentamicin and azithromycin were minimized by curcumin supplementation [112]. The anti-QS compounds, such as gallocatechin 3-gallate and caffeic acid, enhanced therapeutic effects on* Mycoplasma pneumoniae* infection by combining use with tetracycline, ciprofloxacin, or gentamicin [113, 114]. N-(2-pyrimidyl) butylamine was confirmed to enhance the antibacterial effect of tobramycin, colistin, and ciprofloxacin on* Pseudomonas aeruginosa *[115]. Recent studies [116, 117] have shown that both farnesol and hamamelitannin can reduce the virulence of* Staphylococcus aureus* and increase the sensitivity of* Staphylococcus aureus* to *β*-lactam antibiotics. Synergistic effects of hamamelitannin, baicalin, hydrate, cinnamaldehyde, and antibiotics have been demonstrated in different infection models [118, 119]. These findings imply that combining use of antibiotics with ant-QS agents has great therapeutic potential for bacterial diseases.

## 5. Conclusions

Regulating bacterial QS signaling by QS-targeted agents is an effective strategy to control the production of bacterial virulence factors and the formation of biofilm. This novel nonantibiotic therapy can inhibit the expression of pathogenic genes, prevent infection, and reduce the risk of drug resistance of bacterial cells and has been widely exploited in recent years. A large number of studies have identified many anti-QS agents to control the pathogenic phenotypes of most bacteria and to attenuate the pathological damage in various animal infection models. However, most anti-QS agents are still in the preclinical phase and more human clinical trials are warranted to test their practical feasibility. The results of several existing clinical studies [120–122] on anti-QS agents show that, compared with antibiotics, the anti-QS compounds may have potential toxicity and their therapeutic effect is not as stable as that of antibiotics, which limited their extensive application. Combining use of anti-QS agents with conventional antibiotics can significantly improve the efficacy of therapeutic drugs and decrease the cost of human healthcare and is likely to be the main application method of anti-QS agents for bacterial diseases treatment in the future.

## Figures and Tables

**Table 1:** Studies demonstrating the quorum sensing (QS) signaling disruption by receptor inactivation. Abbreviations: 3-oxo-C12, N-3-oxododecanoyl-C12; AHL, N-acyl-homoserine lactones; AI, autoinducer; C4‐LHL, butenyl homoserine lactones; C6‐LHL, hexanoyl homoserine lactones; HSL, L-homoserine lactone; PHL, propionyl homoserine lactones.

Models	Strains	Anti-QS agents	Target	Effects	Ref

*In-vitro*	*Pseudomonas aeruginosa*	Flavonoids	Allosteric inhibition of AI-binding receptors, LasR and RhlR	Altered transcription of QS-controlled target promoters and suppresses virulence factor production	[53]
*In-vitro*	*Pseudomonas aeruginosa *PAO1	N-decanoyl-L-homoserine benzyl ester	Activating quorum sensing control repressor	Attenuated the activity of protease and elastase, swarming motility and biofilm formation	[54, 55]
*In-vitro*, *C. elegans,* A549 cells,	*Pseudomonas aeruginosa* PA14	Meta-bromo-thiolactone	Inhibition of LasR and RhlR	Inhibited both the production of the virulence factor pyocyanin and biofilm formation	[57]
*In-vitro*	*Pseudomonas aeruginosa*	AHL ligands A4, 4-bromophenyl-PHL B7, 4-iodo PHL C10, and 3-nitro PHL C14	Binding to TraR, LasR, and LuxR	Strongly inhibited virulence factor production	[58]
*In-vitro*, Mice	*Aeromonas hydrophila*	C4- and C6-HSLs, 3-oxo-C12-HSL	Regulating the host immune receptor	Increased survivability of infected mice	[59]

**Table 2:** Studies demonstrating the QS disruption by signals synthesis inhibition. Abbreviations: AI, autoinducer; enoyl-ACP, enoyl-acyl carrier protein; HSL, L-homoserine lactone; PHL, propionyl homoserine lactones.

Models	Strains	Anti-QS agents	Target	Effects	Ref

*In-vitro, *Rats	*Streptococcus pneumoniae* D-39	Sinefungin	Inhibition of AI-2 synthesis via downregulating luxS, pfs, and speE expression	Inhibited pneumococcal biofilm growth in vitro and middle ear colonization in vivo	[62]
*In-vitro*	*Pseudomonas aeruginosa*	Sinefungin, butyryl-SAM, and S-adenosylhomocysteine	Inhibiting acyl-HSL signals	Inhibited formation of a covalent acyl–enzyme	[61]
*In-vitro*	*Escherichia coli*	Methylthio-DADMe-immucillin-A	Downregulating 5′-methylthioadenosine, S-adenosyl-homocysteine nucleosidase hydrolyzes	Disrupted key bacterial pathways of methylation, polyamine synthesis, methionine salvage, and quorum sensing	[64]
Mouse	*Plasmodium falciparum*	Triclosan	Inhibiting enoyl-ACP reductase	Protected against blood stages of malaria, enhanced elastic strength	[66]

**Table 3:** Studies demonstrating QS disruption by signals degradation.

Models	Strains	Anti-QS agents	Target	Effects	Ref

*In-vitro*	*Pseudomonas aeruginosa*	Lactonase SsoPox	Degradation of the acyl-homoserine lactones	Inhibited the virulence of 51 clinical P. aeruginosa isolated from diabetic foot ulcers by decreasing the secretion of proteases and pyocyanin, and biofilm formation	[72]
*In-vitro*, rats	*Pseudomonas aeruginosa *PAO1	Lactonase SsoPox-1	Degradation of the acyl-homoserine lactones	Decreased lasB virulence gene activity, pyocyanin synthesis, proteolytic activity, and biofilm formation. Reduced the mortality of rats with acute pneumonia from 75% to 20%. Attenuated lung damage of the rat model	[73]
*In-vitro *	A.baumannii S1,S2,S3	Engineered lactonase	Degradation of the acyl-homoserine lactones	Reduced the biomass of A. baumannii associated biofilms	[74]
*In-vitro *	*Pseudomonas aeruginosa*	Lactonase Aii810	Degradation of the acyl-homoserine lactones	Attenuated Virulence Factors and Biofilm Formation. Degraded N-butyryl-L-homoserine lactone and N-(3-oxododecanoyl)-L-homoserine lactone, by 72.3 and 100%	[75]
*In-vitro *	*Pseudomonas aeruginosa*	Overexpression of lactonase enzyme AHL-1	Degradation of the acyl-homoserine lactones	Reduced the protease, pyocyanin, rhamnolipids. Inhibited the activities on the swarming motility and biofilm formation	[76]
*In-vitro *	*Pseudomonas aeruginosa*	Novel Lactonase cloned by bpiB01, bpiB04	Degradation of the N-(3-oxooctanoyl)-L-homoserine lactone	Inhibited motility and biofilm formation	[77]
*In-vitro *	*Pseudomonas aeruginosa* PAO1	Lactonase AiiK	Degradation of the acyl-homoserine lactones	Inhibited the biofilm formation and attenuates extracellular proteolytic activity and pyocyanin production	[78]
*In-vitro*	*Pseudomonas aeruginosa* PAO1	N-Acyl-Homoserine Lactone Acylase PA2385	Degradation of 3-oxo-C12-HSL and 2-heptyl-3-hydroxy-4(1H)- quinolone	Reduced production of the virulence factors elastase and pyocyanin	[85]
*In-vitro*, *C. elegans*	*Pseudomonas aeruginosa *PAO1	NADP-dependent short-chain dehydrogenase/reductase (bpiB09)	Inactivation of N-(3-oxo-dodecanoyl)-L-homoserine lactone (3-oxo-C12-HSL)	Reduced pyocyanin production, decreased motility, poor biofilm formation and absent paralysis of *C. elegans*	[91]
*In-vitro, *plant	*Pseudomonas aeruginosa *PAO1	3-hydroxy-2-methyl-4(1H)-quinolone	Catalyzing the conversion of PQS to N-octanoylanthranilic acid and carbon monoxide	Reduced expression of the PQS biosynthetic gene pqsA, expression of the PQS-regulated virulence determinants lectin A, pyocyanin, and rhamnolipids, and virulence in plant	[94]

**Table 4:** Applications involving with AHLs degradation by anti-QS agents. 3-oxo-C12, N-3-oxododecanoyl-HSL; AHL, N-acyl-homoserine lactones; C4‐LHL, butenyl homoserine lactones; C6‐LHL, hexanoyl homoserine lactones.

Objectives	Strains	Anti-QS agents	Target	Effects	Ref

Tilapia	*Aeromonas hydrophila*	AHL lactonase AIO6	Degradation of the acyl-homoserine lactones	Maintained the microvilli length in the foregut of tilapia, but significantly lower than those of the control.	[79]
Shrimp and clam	*Vibrionaceae strains*	Deletion of AHLs genes in 34 marine *Vibrionaceae* strains	Acyl-homoserine lactones inactivation	Reduced virulence and mortality of the mutant strains in brine shrimp and Manila clam.	[80]
Shrimp	*Vibrio parahaemolyticus*	AHL-lactonase (AiiA)	Degradation of the acyl-homoserine lactones	Inhibited vibrio biofilm development and attenuated infection and mortality. Reduce vibrio viable counts and biofilm development in the intestine.	[81]
Enzyme multilayer coatings	*Chromobacterium violaceum* CECT 5999, *Pseudomonas aeruginosa* ATCC 10145	Acylase from *Aspergillus melleus*	Degradation of C6‐LHL	Inhibited 50% violacein production by *Chromobacterium violaceum* CECT 5999; Reduces the *Pseudomonas aeruginosa* ATCC 10145 biofilm formation under static and dynamic conditions.	[86]
Acylase‐containing polyurethane coatings	*Pseudomonas aeruginosa *ATCC 10145 and PAO1	Acylase from *Aspergillus melleus*	Degradation of C4‐LHL, C6‐LHL, and 3‐oxo‐C12‐LHL	Immobilization of acylase led to an approximately 60% reduction in biofilm formation, reduce the secretion of pyocyanin.	[87]
Immobilization on Nanofibers	*Pseudomonas aeruginosa* PAO1	Acylase (EC.3.5.1.14)	Degradation of AHL inducers	Reduced the biofilm/biofouling formation under static and continuous flow conditions.	[88]

**Table 5:** Studies demonstrating QS disruption by antibodies targeting.

Models	Strains	Anti-QS agents	Target	Effects	Ref

*In-vitro *and RAW 264.7 cells	*Pseudomonas aeruginosa*	Antibody RS2-1G9 generated against a 3-oxo-dodecanoyl homoserine lactone analog hapten	Targeting the bacterial N-3-oxo-dodecanoyl homoserine lactone molecules	Protect murine bone marrow-derived macrophages from the cytotoxic effects and also prevented the activation of the mitogen-activated protein kinase p38.	[100]
*In-vitro *	*Pseudomonas aeruginosa*	Antibody XYD-11G2	Hydrolyzing N-3-(oxododecanoyl)-L-homoserine lactone	Suppressed QS signaling.	[101]
*In-vitro* and mouse model	*Staphylococcus aureus*	Antibody AP4-24H11 elicited against a rationally designed hapten	Sequestration of the autoinducing peptide-4	Suppressed S. aureus pathogenicity in an abscess formation mouse model *in vivo* and provided complete protection against a lethal *Staphylococcus aureus* challenge.	[103]

**Table 6:** Studies demonstrating the synergistic effects of anti-QS agents and antibiotics.

Models	Strains	Anti-QS agents	Target	Effects	Ref

Mice	*Pseudomonas aeruginosa*	Furanone C-30, ajoene or horseradish juice extract in Combination curcumin	QS inhibition enhance the sensitivity of pathogen to antibiotics	Resulted in an increased clearance of *Pseudomonas aeruginosa* in a foreign-body infection model.	[107]
*In-vitro *	*Pseudomonas aeruginosa*	with tobramycin, gentamicin and azithromycin	Induced concentrations of C12- homoserine lactone and C4- homoserine lactone	Curcumin showed synergistic effects with azithromycin and gentamicin. Combination use reduced QS-related virulence factors. Downregulated QS-related genes.	[112]
*In-vitro*	*Staphylococci*	Epigallocatechin-3-gallate with Tetracycline	Inhibition of the activity of Tet(K) pumps efflux pumps of a different class Tet(B)	Enhanced the bactericidal effect of Tetracycline on *Staphylococci.*	[113]
*In-vitro*	*Pseudomonas aeruginosa*	N-(2-pyrimidyl) butanamide, C11	Downregulation of rhl, rhlA and lasB genes	Increased the susceptibility to antibiotics and attenuated the pathogenicity of the bacterium.	[115]
*In-vitro*	*Staphylococcus aureus* (methicillin-resistant *S. aureus*)	Farnesol with *β*-lactam antibiotics	Inhibition of lipase activity and disruption of the cytoplasmic membrane through the leakage of potassium ions	Attenuated the rate of growth of bacteria, and countering ubiquitous *β*-lactam resistance in bacteria.	[116, 117]
*In-vitro*, *C. elegans*, *Galleria mellonella*, mice	*Burkholderia cenocepacia*, *Staphylococcus aureus*, *Escherichia coli*	Baicalin hydrate, cinnamaldehyde, hamamelitannin with Tobramycin, vancomycin, and clindamycin;	Inhibition of biofilm formation. QS inhibition enhance the sensitivity of pathogen to antibiotics	Combining the use of antibody and anti-QS agents increased susceptibility of the bacteria to the antibiotic, and increased host survival rate after infection	[118]
